# A 34-Marker Panel for Imaging Mass Cytometric Analysis of Human Snap-Frozen Tissue

**DOI:** 10.3389/fimmu.2020.01466

**Published:** 2020-07-16

**Authors:** Nannan Guo, Vincent van Unen, Marieke E. Ijsselsteijn, Laura F. Ouboter, Andrea E. van der Meulen, Susana M. Chuva de Sousa Lopes, Noel F. C. C. de Miranda, Frits Koning, Na Li

**Affiliations:** ^1^Immunohematology and Blood Transfusion, Leiden University Medical Center, Leiden, Netherlands; ^2^Institute for Immunity, Transplantation and Infection, Stanford University, Stanford, CA, United States; ^3^Pathology, Leiden University Medical Center, Leiden, Netherlands; ^4^Gastroenterology, Leiden University Medical Center, Leiden, Netherlands; ^5^Anatomy, Leiden University Medical Center, Leiden, Netherlands; ^6^Key Laboratory of Zoonoses Research, Ministry of Education, Institute of Zoonoses, College of Veterinary Medicine, Jilin University, Changchun, China

**Keywords:** imaging mass cytometry, IMC, snap-frozen tissue sections, human intestine, mass cytometry

## Abstract

Imaging mass cytometry (IMC) is able to quantify the expression of dozens of markers at sub-cellular resolution on a single tissue section by combining a novel laser ablation system with mass cytometry. As such, it allows us to gain spatial information and antigen quantification *in situ*, and can be applied to both snap-frozen and formalin-fixed, paraffin-embedded (FFPE) tissue sections. Herein, we have developed and optimized the immunodetection conditions for a 34-antibody panel for use on human snap-frozen tissue sections. For this, we tested the performance of 80 antibodies. Moreover, we compared tissue drying times, fixation procedures and antibody incubation conditions. We observed that variations in the drying times of tissue sections had little impact on the quality of the images. Fixation with methanol for 5 min at −20°C or 1% paraformaldehyde (PFA) for 5 min at room temperature followed by methanol for 5 min at −20°C were superior to fixation with acetone or PFA only. Finally, we observed that antibody incubation overnight at 4°C yielded more consistent results as compared to staining at room temperature for 5 h. Finally, we used the optimized method for staining of human fetal and adult intestinal tissue samples. We present the tissue architecture and spatial distribution of the stromal cells and immune cells in these samples visualizing blood vessels, the epithelium and lamina propria based on the expression of α-smooth muscle actin (α-SMA), E-Cadherin and Vimentin, while simultaneously revealing the colocalization of T cells, innate lymphoid cells (ILCs), and various myeloid cell subsets in the lamina propria of the human fetal intestine. We expect that this work can aid the scientific community who wish to improve IMC data quality.

## Introduction

In recent years, the development of a variety of single-cell technologies increased recognition of cellular heterogeneity both in physiological and pathological contexts. Single-cell technologies based on RNA sequencing and mass cytometry (CyTOF) have been utilized to investigate cellular heterogeneity and identify novel cellular subsets (1, 2), and to discover biomarkers with clinical value (3). Single-cell mass cytometry employs antibodies conjugated to stable metal isotopes, mostly from the lanthanide series, and is currently able to analyze over 40 different markers simultaneously, allowing an in-depth analysis of immune subsets. However, when analyzing cells isolated from tissue, no spatial information on cell-cell interactions within the tissue is obtained. Imaging mass cytometry (IMC) is an extension of mass cytometry, which couples a laser ablation system with a mass cytometer (4) and therefore has the ability to analyze up to 40 markers in a single tissue section. As such, IMC has the potential to simultaneously characterize the composition of the immune compartment, the spatial relationship between immune cells and stromal cells, and the interactions among immune subsets in tissue sections of choice.

Classical immunohistochemistry or immunofluorescence techniques for cell and tissue imaging provide high spatial resolution at subcellular resolution (5), however, these suffer from limitations including the limited number of markers that can be used simultaneously and tissue auto-fluorescence (6). IMC does not suffer from background interference as the read-out is provided by the presence of rare earth metals conjugated to antibodies which considerably increase the multiplexing capacity. The IMC laser system ablates the tissue in segments of one by one micrometer which are directed into the mass cytometer using a gas stream, then atomized and ionized followed by determination of the metal-isotope ion content in the on-line time-of-flight mass analyzer (7). IMC thus offers significant advantages over the current imaging standards. However, care should be taken with the design of the antibody panels as there can be spillover detectable from one mass channel into other channels due to isotopic impurities of the rare metals, usually below 3% (8), and a method has been developed to reduce spillover artifacts and improve the generation of high-quality data (9). IMC is rapidly becoming widespread as it can aid both basic research and clinical practice (10, 11).

However, the use of IMC is still challenging due to the limited experience with the design and validation of antibody panels and the best tissue processing procedures and staining procedures compatible with the dozens of antibodies that are applied simultaneously, especially with respect to snap-frozen tissue as most experience to date is with formalin-fixed paraffin-embedded (FFPE) tissue.

Here, we developed a 34 antibody panel for the analysis of snap-frozen tissues by IMC, which contains immune lineage and additional markers to distinguish immune cell subsets in addition to structural markers to reveal tissue organization. This panel can be used to obtain comprehensive spatial information on interactions both between immune cell subsets and between immune cell subsets and stromal components. Furthermore, we developed an optimized fixation and antibody incubation protocol to improve the IMC data quality. We anticipate that this optimized methodology will give guidance to the scientific community in using IMC on snap-frozen tissue to generate high-quality images.

## Materials and Methods

### Tissue Samples

Fetal tissues were obtained from elective abortions with informed consent. The adult intestinal samples were collected from patients undergoing routine diagnostic endoscopies. Approval by the medical ethical commission of the Leiden University Medical Center (protocol P08.087) was obtained in accordance with the local ethical guidelines and the Declaration of Helsinki. The adult and fetal intestinal samples were embedded in optimal cutting temperature compound, snap-frozen in isopentane (VWR) and stored at −80°C.

### Antibody Validation and Conjugation

Antigens were selected based on previously published single-cell mass cytometry and single-cell RNA sequencing data on the human fetal intestinal samples (1, 12, 13). Antibodies used for IMC are listed in Table 1. 16 of the 34 antibodies used in the current panel were directly purchased from Fluidigm, which were already conjugated with metals. For the remaining 18 antibodies, BSA-free and carrier-free formulations of antibodies were purchased from different suppliers and initially tested for performance by immunohistochemical staining (IHC) on human fetal intestine and tonsil. Subsequently, antibodies with an appropriate signal intensity were conjugated to lanthanide metals using the MaxPar Antibody Labeling Kit (Fluidigm) following the manufacturer's instructions. Post-conjugation, all antibodies were eluted in 100 μl W-buffer (Fluidigm) and 100 μl antibody stabilizer buffer (Candor Bioscience, Wangen im Allgäu, Germany) supplemented with 0.05% sodium azide.

**Table 1:** The 34-marker panel for imaging mass cytometry on snap-frozen tissue.

	**Antigen**	**Tag**	**Clone**	**Supplier**	**Cat**.	**Dilution**
1	CD45	89Y	HI30	Flui	3089003B	1/50
2	D2-40	115In	D2-40	BioL	916606	1/50
3	FOXp3	142Nd	D608R	CST	12653BF	1/50
4	CD69	144Nd	FN50	Flui	3144018B	1/50
5	CD4	145Nd	RPA-T4	Flui	3145001B	1/50
6	CD8a	146Nd	RPA-T8	Flui	3146001B	1/50
7	Collagen I	147Sm	Polyclonal	Millipore	AB758	1/100
8	α-SMA	148Nd	1A4	CST	CST5685BF	1/200
9	CD31	149Sm	8 9C2	CST	CST3528BF	1/100
10	E-Cadherin	150Nd	24 E 10	CST	CST3195BF	1/50
11	CD123	151Eu	6H6	Flui	3151001B	1/50
12	CD7	153Eu	CD7-6B7	Flui	3153014B	1/100
13	CD163	154Sm	GHI/61	Flui	3154007B	1/100
14	CD103	155Gd	EPR4166	Abcam	ab221210	1/50
15	CD127	156Gd	R34.34	Beckman	18LIQ494	1/50
16	CD122	158Gd	TU27	BioL	339015	1/25
17	CD68	159Tb	KP1	Flui	3159035D	1/200
18	CD5	160Gd	UCHT2	BioL	300627	1/25
19	CD20	161Dy	H1	Flui	3161029D	1/50
20	CD11c	162Dy	Bu15	Flui	3162005B	1/50
21	CD45	163Dy	D9M81	CST	13917BF	1/200
22	CD161	164Dy	HP-3G10	Flui	3164009B	1/50
23	CD117	165Ho	104D2	BioL	313202	1/50
24	Ki-67	166Er	D3B5	CST	CST 9129BF	1/200
25	CD27	167Er	O323	Flui	3167002B	1/50
26	HLA-DR	168Er	L243	BIoL	307651	1/800
27	CD45RA	169Tm	HI100	Flui	3169008B	1/100
28	CD3	170Er	UCHT1	Flui	3170001B	1/100
29	CD28	171Yb	CD28.2	BioL	302937	1/50
30	CD38	172Yb	HIT2	Flui	3172007B	1/100
31	CD45RO	173Yb	UCHL1	BioL	304239	1/50
32	CD57	174Yb	HNK-1/Leu-7	Abcam	Ab212403	1/100
33	Vimentin	175Lu	D21H3	CST	CST5741BF	1/200
34	CD56	176Yb	NCAM16.2	Flui	3176008B	1/50

*Flui, Fluidigm; CST, cell signaling technology; Biol, Biolegend*.

### Optimization of IMC Immunostaining Protocol

Here, three variables were tested: (1) Drying condition of freshly prepared snap-frozen tissue sections; (2) Fixation procedures; and (3) Antibody staining conditions. For drying we compared 3 min at room temperature (RT) with 30 min at RT, and 1 h at 60°C. For fixation we compared methanol for 5 min at −20°C, with 1% PFA for 5 min at RT, 1% PFA for 5 min at RT followed by methanol for 5 min at −20°C, acetone for 10 min at RT, and 4% PFA for 5 min at RT. For antibody incubation we compared 5 h at RT with overnight at 4°C. We utilized one frozen sample to test each condition and a single antibody mix to stain all section slides. An overview of the experimental set up for the testing of the various conditions is provided in Table 2. All comparisons were performed simultaneously. The following is a step-by-step staining procedure of the IMC procedure utilizing snap-frozen tissue.

**Table 2:** The experimental set up of the testing of the various conditions.

**Slide Nr**	**Slide drying**			**Fixation**					**Panel incubation**	
	**3 min**	**30 min**	**1 h**	**Methanol**	**1% PFA**	**1% PFA + methanol**	**Acetone**	**4% PFA**	**5 h at RT**	**Overnight at 4^**°**^C**
1	+	–	–	–	–	+	–	–	–	+
2	–	+	–	+	–	–	–	–	–	+
3	–	+	–	–	+	–	–	–	–	+
4	–	+	–	–	–	+	–	–	–	+
5	–	+	–	–	–	–	+	–	–	+
6	–	+	–	–	–	–	–	+	–	+
7	–	+	–	–	–	+	–	–	+	–
8^*^	–	–	+	–	–	+	–	–	–	+

* *Conditions applied to slide #8 represent the optimal staining protocol*.

### Material

- Five micrometer fresh snap-frozen sections on silane-coated glass slides (VWR)
- Paraformaldehyde (1%, 4%)
- Methanol
- Acetone
- Superblock solution (Thermo Fisher Scientific)
- DPBS (Gibco)
- Wash buffer (DPBS supplemented with 0.05% Tween and 1% BSA)
- Metal-conjugated antibodies (Table 1)
- Intercalator-Ir (500 μM, Fluidigm)
- Milli-Q water
- Dako Pen (Thermo Fisher Scientific)
- Slide container, 5 slide capacity (VWR)
- Incubation chamber (humid, 4°C and RT).

### Stepwise Procedure for Immunodetection

1. Cut the fresh frozen sections at 5 μm and mount them on silane-coated glass slides
2. Dry the tissue sections for 3 min at RT, 30 min at RT or 1 h at 60°C
3. Fix the tissue slides without shaking as mentioned above
4. Rinse the slides once, followed by washing the slides twice for 5 min in a container of 5 slide capacity with 25 ml wash buffer
5. Rehydrate the slides for 5 min in container of 5 slide capacity with 25 ml DPBS
6. Wash the slides for 5 min in container of 5 slide capacity with 25 ml wash buffer
7. Use the Dako Pen to draw a circle around the tissue sections to create a barrier to contain the antibody solutions on the tissue sections
8. Apply 100 μl superblock solution to each slide for 30 min at RT
9. Remove excess superblock solution by tapping on a tissue
10. Prepare the antibody cocktail by diluting the antibodies in wash buffer as described in Table 1
11. Add 100 μl of the antibody cocktail to each section and incubate for 5 h at RT or overnight at 4°C in a humid chamber
12. After the incubation, wash the sections three times for 5 min in container of 5 slide capacity with 25 ml wash buffer
13. Incubate the slides with 100 μl 1:400 dilution of Intercalator-Ir in DPBS for 30 min at RT
14. Rinse the slides once, wash the slides for 5 min in container of 5 slide capacity with 25 ml wash buffer twice
15. Wash the slides for 1 min in container of 5 slide capacity with 25 ml Milli-Q water
16. Dry the slides with an air flow
17. Store the slides at 4°C until ablation on Hyperion.

### Imaging Mass Cytometry Acquisition

Tissue acquisition was performed on a Helios time-of-flight mass cytometer coupled to a Hyperion Imaging System (Fluidigm). All IMC operation was performed as described using the Hyperion Imaging System (Fluidigm). Briefly, after flushing the ablation chamber with helium, tissues were ablated by a UV-laser spot-by-spot at a resolution of 1 μm and a frequency of 200 Hz. Regions of interest (ROIs) with 1,000 μm × 1,000 μm were selected. We ablated 5~8 ROIs for each tissue section. All raw data were analyzed for marker intensity based on the maximum signal threshold, defines at the 98th percentile of all pixels in a single ROI using the Fluidigm MCD^TM^ viewer (v1.0.560.2). To distinguish the signal from background, we used the Fluidigm MCD^TM^ viewer to visualize our data, and adjusted the Threshold Min values for each marker individually (between 1 and 2 for majority of immune markers and between 1 and 3 for structural markers) to eliminate background.

## Results

To develop the IMC antibody panel, we first evaluated the performance of an antibody panel previously developed for cell suspension mass cytometry (1). This revealed that 18 out of the 36 antibodies were suitable for IMC on snap-frozen tissue. Subsequently we continued to select additional antibodies to phenotype immune cells and visualize the tissue structure. All candidate antibodies which required in-house conjugation with metals were initially tested for performance by conventional immunohistochemistry (not shown). Based on this we selected antibodies that displayed a clear signal-to-background ratio for potential inclusion in the final IMC antibody panel. In total, 80 antibodies were tested, 43 of which performed well on frozen sections. Table 2 lists the 34 antibodies that were finally chosen for inclusion into the IMC antibody panel. Supplementary Table 1 provides information on the performance of the 46 antibodies that were not included in the panel.

In order to ensure proper tissue adherence, we determined the influence of the time of drying for the freshly prepared tissue sections. We evaluated the staining obtained with each of the 34 antibodies on tissue sections that were either dried for 3 or 30 min at RT, or for 1 h at 60°C. Both visual inspection of the obtained images and comparison of the maximum signal threshold values for each antibody indicated that the staining intensity was comparable with all three drying conditions (Supplementary Figure 1A). We also observed that the signal-to-background ratio was highly similar with the three tested conditions (Supplementary Figure 1B). Therefore, we conclude that the drying conditions tested are in principle all suitable for IMC on snap-frozen tissue sections.

**Figure 1:**
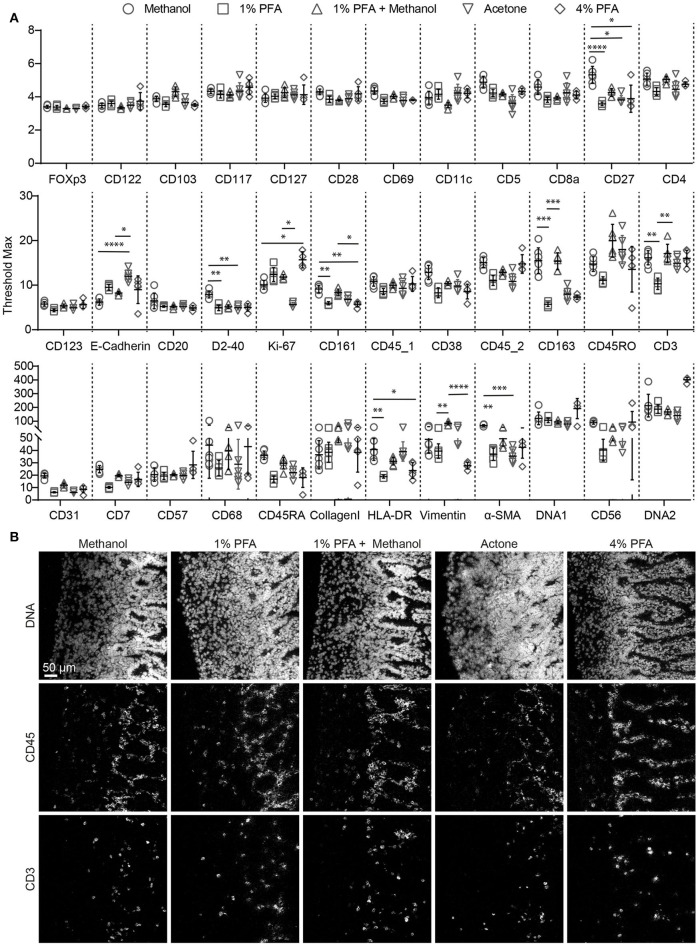
Comparison of antibody and nuclear staining between different fixation procedures for IMC within a single tissue block. **(A)** Comparison of the staining intensity of each antibody depending on the fixation conditions, based on the maximum signal threshold in MCD^TM^ viewer. Black bars indicate median ± IQR. Each gray dot represents an individual ROI. *P < 0.05, **P < 0.01, ***P < 0.001 and ****P < 0.0001 by Kruskal-Wallis test with Dunn's test for multiple comparisons. **(B)** The markers CD45 and CD3 are representative for the variations observed with the tested incubation conditions. The minimum signal threshold of 2 dual count was set for the nuclear staining, while that was 1.5 for the immune markers.

As tissue fixation is required to preserve antigenic determinants in tissues we first evaluated the protocol provided by Fluidigm (14). However, we observed that acetone fixation did not yield satisfactory results with respect to the quality of both the nuclear staining and the antibody staining (not shown). Therefore, we proceeded to test additional fixation procedures to optimize signal intensity and signal-to- background ratio. We tested 5 conditions, using serial sections from a single tissue sample: methanol, 1% PFA, 1% PFA followed by methanol, acetone and 4% PFA and evaluated the staining obtained with the 34 antibodies individually (Figures 1A,B). We observed that none of the tested fixation conditions yielded optimal results for all antibodies in the panel. As expected, we observed inadequate nuclear staining with acetone, incompatible with proper cell identification and cell segmentation analysis (Figure 1B). Moreover, comparison of the maximum signal threshold values for each antibody indicated that several markers performed relatively poor when either 1 or 4% PFA were used for fixation (e.g., CD161, CD163, CD3, CD7, CD68, HLA-DR, Vimentin, α-SMA) while fixation with methanol or 1% PFA followed by methanol yielded stronger signals. In addition, we observed higher background staining for immune markers (e.g., anti-CD45, clone HI30, and anti-CD3, Figure 1B) in both the acetone and PFA-only samples while the methanol and 1% PFA followed by methanol fixed samples provided superior antibody staining results (Figure 1B). However, the nuclear staining in the lamina propria of the intestine was slightly better in the PFA + methanol samples. Based on these observation, we conclude that fixation with methanol or with the combination of 1% PFA followed by methanol are both preferred for IMC immunodetection of snap-frozen samples.

**Figure 2:**
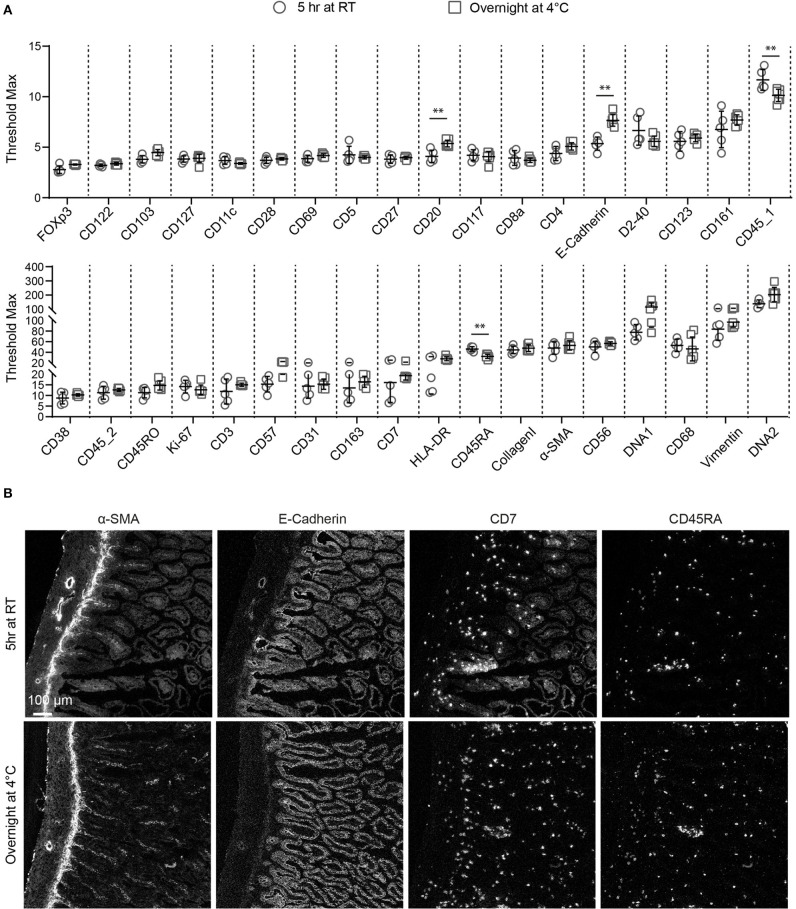
Comparison of antibody performance between two immunodetection conditions for IMC. **(A)** The staining intensity of each antibody at either 5 h at room temperature or overnight at 4°C is shown, based on the maximum signal threshold in MCD^TM^ viewer. Black bars indicate median ± IQR. Each gray dot represents an individual ROI. **P < 0.01 by Mann-Whitney U test. **(B)** The structural markers E-Cadherin, α-SMA and the immune markers CD7, CD45RA are representative for the variations observed with the tested conditions.

As staining quality is strongly influenced by duration of and temperature during antibody incubation (15), we tested two different incubation conditions for the individual antibodies in the 34-marker panel: 5 h at RT or overnight at 4°C, after which the signal intensity and specificity were assessed by IMC for each antibody. We also determined the maximum signal threshold for all antibodies within several ROIs to compare the staining intensity between the two conditions. We found that the staining intensity of many antibodies were similar under both conditions, while a number of markers performed better either at 4°C (anti-CD20 and anti-E-Cadherin) or at RT (anti-CD45_1, and anti-CD45RA) (Figure 2A). However, we observed more variation in the maximum threshold values for the evaluated ROIs stained at RT compared to 4°C and for many antibodies higher background was observed at RT. For example, anti-α-SMA, anti-E-Cadherin and anti-CD7 yielded higher specific staining and lower background after overnight incubation at 4°C compared to a 5 h incubation at RT while several other antibodies performed equally well at both test conditions as observed with anti-CD45RA (Figure 2B). As incubation at 4°C yielded generally better results we decided to use this condition for validation of the full antibody panel.

**Figure 3:**
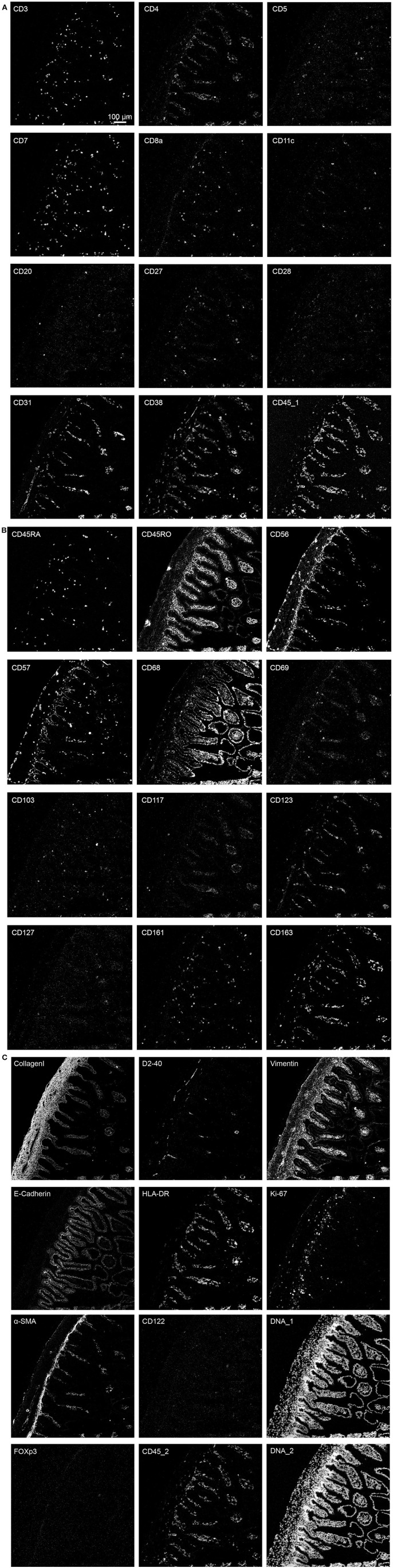
**(A–C)** The optimized immunodetection of the 34-marker panel and nuclear staining in a single representative ROI for IMC on the human fetal intestine.

We next applied the optimized protocol in which the tissue section was dried for 1 h at 60°C, followed by fixation with PFA + methanol and antibody panel incubation overnight at 4°C to stain a human fetal intestinal sample with the full 34-antibody panel which included structural tissue markers (Collagen I, E-Cadherin, α-SMA, Vimentin and D2-40) as well as markers to identify various cell types within the lymphoid and myeloid compartments (Table 1). Moreover, the panel allows for the visualization of additional features such as naïve and memory states (CD45RA/RO), cell division (Ki-67), tissue-residency (CD103 and CD69), and expression of cytokine receptors (e.g., CD122 and CD127) (Figures 3A–C).

**Figure 4:**
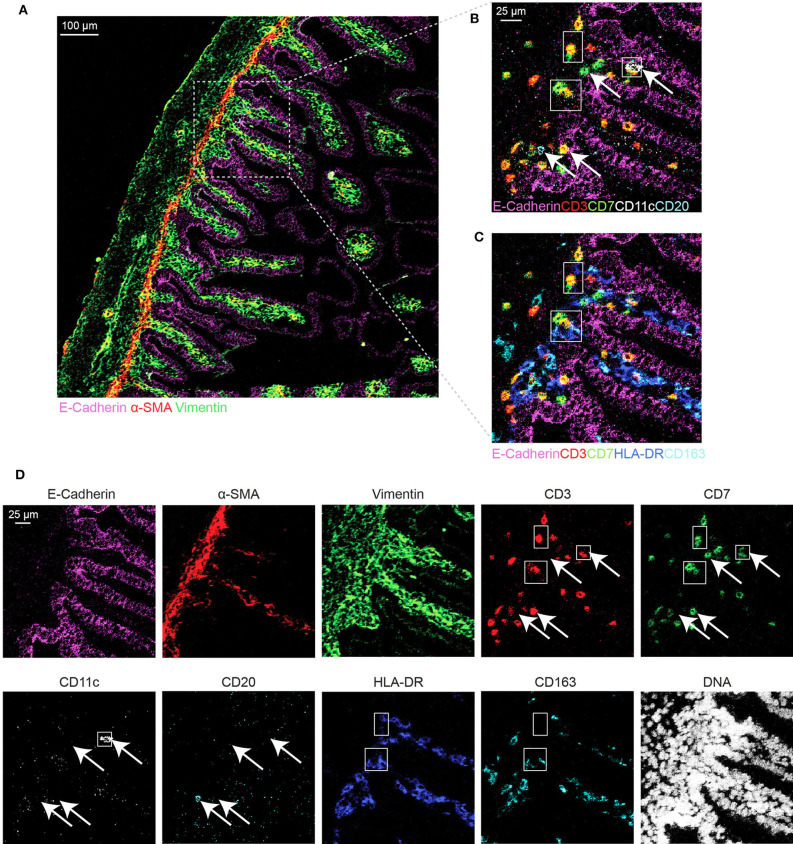
Visualization of the tissue structure and detection of immune cell types in a single region of interest in the human fetal intestine by IMC. **(A)** Representative mass cytometry image of the fetal intestine showing the overlay of E-Cadherin (magenta), Vimentin (green), and α-SMA (red). **(B–D)** Identification of immune cell subsets. **(B)** T cells (CD3^+^CD7^+^), innate lymphoid cells (ILCs, CD3^+^CD7^−^) and B cells (CD20^+^); **(C)** myeloid cell (CD11c^+^) and macrophages (HLA-DR^+^CD163^+^). The arrows indicate different immune cell types, while the boxes indicate the interaction between ILCs, T cells, and myeloid cells. **(D)** Individual antibody stains.

**Figure 5:**
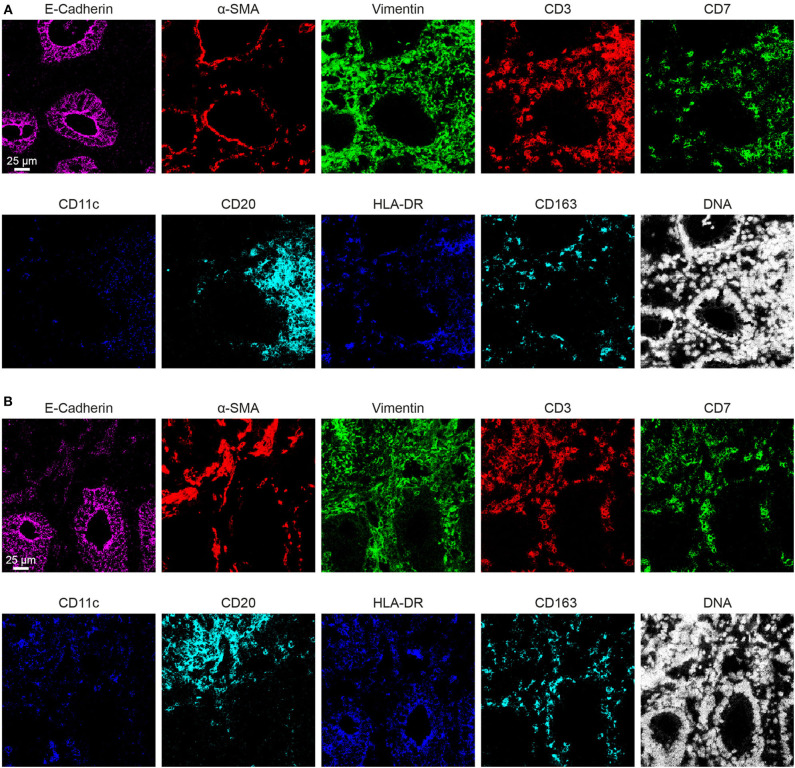
Representative mass cytometry images of 2 adult intestines. **(A)** The adult intestinal sample from a healthy control. **(B)** The adult intestinal sample from a patient with inflammatory bowel disease.

Based on the adjusted Threshold Min and default Threshold Max in the MCD^TM^ viewer (Table 3), the resulting images were analyzed. Collagen I immunodetection was used to delineate the extracellular matrix of the basement membrane which exhibited the highest staining intensity (Figure 3C). Vessels with smooth muscle lining were detected by the presence of α-smooth muscle actin (α-SMA, Figures 3C, 4A), and CD31 and D2-40 staining (Figures 3A,C). The epithelium and lamina propria were distinguished as Vimentin^−^E-Cadherin^+^ and Vimentin^+^E-Cadherin^−^, respectively (Figure 4A). Cells of hematopoietic origin were identified with an anti-CD45 specific antibody, revealing that the majority of the immune cells were localized in the lamina propria (Figure 3). To define the spatial distribution of different immune subsets in the human fetal intestine, T cells (CD3^+^CD7^+^), innate lymphoid cells (ILCs, CD3^−^CD7^+^), B cells (CD20^+^), CD11c^+^HLA-DR^+^ myeloid cells, and macrophages (HLA-DR^+^CD163^+^), were identified and visualized in a single region of interest (Figures 4B,C). For comparison, the individual stains for DNA, the structural markers E-Cadherin, α-SMA, and Vimentin, as well as the immune markers CD3, CD7, CD20, CD11c, HLA-DR, and CD163 are shown in Figure 4D. In Figure 4B a single CD20^+^ B cells is identified (cyan) while CD3^+^CD7^+^ T cells (yellow) and CD3^−^CD7^+^ ILCs (green) are present both as isolated cells and adjacent to each other (two boxed areas on the left side of the image, Figure 4B). In addition, a white CD11c^+^ myeloid cells was detected colocalized with a T cell (boxed area on the right side of the image, Figure 4B). Moreover, the visualization of HLA-DR and CD163 reveals the close association of HLA-DR^+^CD163^+^ macrophages (blue/cyan) with adjacent T cells and ILCs (two boxed area's on the left side of the image, Figure 4C), and several clusters of T cells and HLA-DR^+^ myeloid cells (Figure 4C). Thus, the optimized approach for snap-frozen tissue analysis with IMC presented here facilitates the simultaneous identification of multiple distinct cells types and distinct colocalization patterns thereof in a single image. In addition we applied the optimized staining protocol with the full antibody panel to two adult intestinal samples, one from a healthy control (Figure 5A) and another from a patient with inflammatory bowel disease (Figure 5B). Here we observed clear tissue structures based on E-Cadherin, α-SMA, Vimentin, and DNA staining (Figure 5). Moreover, visualization of the immune lineage markers CD3, CD7, CD20, HLA-DR, CD163, and CD11c revealed the presence and distribution of lymphoid and myeloid immune cell subsets within the tissue context in a single section (Figure 5).

**Table 3:** The estimated signal-to- background ratio of the antibodies in the optimal staining protocol.

**Antigen**	**Channel**	**Adjusted threshold min**	**Default threshold max**	Estimated signal-to-background ratio*
CD3	170Er	1.00	17.94	17.94
CD4	145Nd	1.50	5.41	3.61
CD5	160Gd	1.50	4.17	2.78
CD7	153Eu	1.50	21.97	14.65
CD8a	146Nd	1.50	4.60	3.07
CD11c	162Dy	1.00	4.42	4.42
CD20	161Dy	2.00	5.08	2.54
CD27	167Er	1.00	4.93	4.93
CD28	171Yb	1.00	3.99	3.99
CD31	149Sm	2.00	18.62	9.31
CD38	172Yb	1.50	12.01	8.01
CD45_1	89Y	1.50	9.66	6.44
CD45RA	169Tm	2.00	32.74	16.37
CD45RO	173Yb	3.00	17.60	5.87
CD56	176Yb	5.00	61.80	12.36
CD57	174Yb	5.00	22.25	4.45
CD68	159Tb	5.00	66.47	13.29
CD69	144Nd	1.50	4.20	2.80
CD103	155Gd	1.50	4.16	2.77
CD117	165Ho	1.00	4.58	4.58
CD123	151Eu	1.50	6.46	4.31
CD127	156Gd	2.00	4.32	2.16
CD161	164Dy	1.50	7.42	4.95
CD163	154Sm	2.00	23.10	11.55
Collagen I	147Sm	3.00	46.60	15.53
D2-40	115In	3.00	6.56	2.19
Vimentin	175Lu	5.00	94.23	18.85
E-Cadherin	150Nd	1.50	7.89	5.26
HLA-DR	168Er	3.00	41.36	13.79
Ki-67	166Er	2.00	11.69	5.85
α-SMA	148Nd	3.00	46.20	15.40
CD122	158Gd	1.00	3.48	3.48
DNA1	191Ir	3.00	44.85	14.95
FOXp3	142Nd	2.00	3.10	1.55
CD45_2	163Dy	2.00	14.10	7.05
DNA2	193Ir	3.00	76.37	25.46

* *Estimated Signal-to-background was defined as Default Threshold Max/Adjusted Threshold Min*.

## Discussion

We report the development of a 34-antibody panel and an optimized staining protocol for snap-frozen tissue sections for analysis with IMC. Based on staining intensity and signal-to- background ratio, we compared different fixation procedures, drying time of the tissue sections, and the impact of duration and temperature during the antibody incubation. In principle, IMC is applicable to both FFPE and snap-frozen tissue but most studies so far have used FFPE tissue. In contrast to FFPE, snap-frozen tissue samples do not require antigen retrieval, thus simplifying the immunodetection protocol. Moreover, antibodies that can be used with frozen tissue cannot always be employed with FFPE tissue and vice versa. Thus, it is useful to have both options available. Previously, Chang et al. (14) have shown that acetone can be used for the fixation of frozen and FFPE tissue for IMC. However, while we also observed that acetone fixation can be used, the low quality DNA staining did not allow an optimal cell segmentation analysis. Therefore, we compared several fixation procedures that identified methanol or a combination of 1% PFA and methanol as appropriate for snap-frozen samples. While the tested variations in drying condition of the tissue samples did not influence the outcome of the staining procedure, we observed that antibody incubation overnight at 4°C yielded optimal results.

We applied the 34-antibody panel to identify various stromal elements and a variety of immune cell subsets in the human fetal intestine. The localization of collagen I, Vimentin, E-Cadherin and α-SMA allowed the visualization of the major architecture of the tissue sample and distinction of the villi, crypts, basal membrane and lamina propria. Simultaneously, T cells, ILCs, and various myeloid cell subsets could be identified as well as interactions between these cell types individually and in clusters of lymphoid and myeloid cells. Here, the specific co-localization of ILCs, T cells, and myeloid cells in the lamina propria suggests that the ILC may somehow modulate the interaction between the T cells and myeloid cells directly. Moreover, recent findings have shown that memory T cells are generated in the human fetal intestine and the specific co-localization of T cells and myeloid cells may ultimately reveal where such memory responses are initiated (16). Here, additional markers in the antibody panel, like HLA-DR and Ki-67, will likely aid in the identification of activated T cells *in situ*.

In the present study we have used the MCD^TM^ viewer software to visualize the images of the tissue sections. In addition cell segmentation approaches based on the identification of nuclei have been developed to aid in the visualization of IMC data (17, 18) as well as computational approaches to identify and quantify cell-cell interactions like Imacyte and Histocat (19, 20). Together this allows for an in depth investigation of cellular interactions in a variety of tissues. Thus, IMC offers a major advantage over classical immunohistochemistry techniques which are limited by the numbers of markers that can be included simultaneously. Together with other studies that have developed antibody panels for FFPE tissue (15, 21, 22) this sets the stage for detailed studies to determine immune heterogeneity and cell-cell interactions *in situ*, providing a novel layer of understanding of functioning of the immune system on tissues. We anticipate that our study will guide other researchers that wish to use IMC for analysis of tissue of choice. Here the conditions defined in the present study can be used as a starting point, however, we like to emphasize that every tissue has its own characteristics that may require further optimization for the tissue under investigation.

## Data Availability Statement

All datasets presented in this study are included in the article/Supplementary Material.

## Ethics Statement

The studies involving human participants were reviewed and approved by Leiden University Medical Center_LUMC (Protocol P08.087). The patients/participants provided their written informed consent to participate in this study. Written informed consent was obtained from the individual(s) for the publication of any potentially identifiable images or data included in this article.

## Author Contributions

NG, NL, and FK conceived the study and wrote the manuscript. NG performed most experiments with the help of VU. NG performed most of the analyses with the help of VU, MI, LO, AM, and NM. SC provided human fetal tissues. All authors discussed the results and commented on the manuscript.

## Conflict of Interest

The authors declare that the research was conducted in the absence of any commercial or financial relationships that could be construed as a potential conflict of interest.
