# Supplementary material for: A 34-Marker Panel for Imaging Mass Cytometric Analysis of Human Snap-Frozen Tissue
Source: Front Immunol. 2020 Jul 16;11:1466. doi: 10.3389/fimmu.2020.01466 (PMC7381123; doi:10.3389/fimmu.2020.01466)
Supplement: Supplementary file 1 [file Data_Sheet_1.docx]

Supplementary Material

**Supplementary table 1** Antibody validation for exclusion in the 34-antibody panel for IMC on snap-frozen tissue sections.

|  | **Antigen** | **Tag** | **Clone** | **Supplier** | **Cat.** | **Suitable for IMC** |
| --- | --- | --- | --- | --- | --- | --- |
| 1 | CD1a | 115In | 010 | Dako | M357101-2 | Yes |
| 2 | CD24 | 198Pt | ML5 | BD eBioscience | 555426 | Yes |
| 3 | CD26 | 157Gd | BA5b | Biolegend | 302702 | Yes |
| 4 | CD30 | 158Gd | Ber-H2 | Dako | M075101-2 | Yes |
| 5 | FcεRIα | 143Nd | AER-37 (CRA-1) | Biolegend | 334602 | Yes |
| 6 | FOXp3 | 142Nd | D608R | Cell Signaling Technology | 12653BF | Yes |
| 7 | ICOS | 143Nd | D6N8TYM | Cell Signaling Technology | CST89601BF | Yes |
| 8 | SOX6 | 158Gd | polyclonal | Abcam | ab30455 | Yes |
| 9 | TCRαβ | 141Pr | 8A3 | Thermo Fisher | TCR1151 | Yes |
| 10 | CCR6 | 141Pr | G034E3 | Fluidigm | 3141003A | No |
| 11 | CCR7 | 115In | Y59 | Abcam | ab221209 | No |
| 12 | CCR7 | 159Tb | G043H7 | Fluidigm | 3159003A | No |
| 13 | CD103 | 155Gd | Ber-ACT8 | Biolegend | 350202 | No |
| 14 | CD11b | 209Bi | ICRF44 | Fluidigm | 3209003B | No |
| 15 | CD127 | 165Ho | AO19D5 | Fluidigm | 3165008B | No |
| 16 | CD127 | 168Er | EPR2955(2) | Abcam | ab180521 | No |
| 17 | CD14 | 160Gd | M5E2 | Fluidigm | 3160001B | No |
| 18 | CD14 | Qdot800 | TüK4 | Thermo Fisher | Q10064 | No |
| 19 | CD142 | 194Pt | TF9-10H10 | Thermo Fisher | MA1-83495 | No |
| 20 | CD15 | 115In | W6D3 | Biolegend | 323035 | No |
| 21 | CD16 | 148Nd | 3G8 | Fluidigm | 3148004B | No |
| 22 | CD1a | 142Nd | HI149 | Sony Biotechnology | 2100510 | No |
| 23 | CD20 | 163Dy | 2H7 | Biolegend | 302343 | No |
| 24 | CD25 | 149Sm | 2A3 | Fluidigm | 3149010B | No |
| 25 | CD34 | 142Nd | 581 | Biolegend | 343531 | No |
| 26 | CD74 | 157Gd | LN2 | BD eBioscience | 555612 | No |
| 27 | CD8b | 166Er | SIDI8BEE | BD eBioscience | 14-5273 | No |
| 28 | CD90 | 104Pt | 5E10 | BD eBioscience | 14-0909-82 | No |
| 29 | C-Kit | 143Nd | 104D2 | Fluidigm | 3143001B | No |
| 30 | C-Kit | 143Nd | D3W6Y | Fluidigm | 37805BF | No |
| 31 | CRTH2 | 156Gd | BM16 | Biolegend | 350102 | No |
| 32 | Eomes | 165Ho | 21Mags8 | BD eBioscience | 14-4876-82 | No |
| 33 | Granzyme B | 141Pr | EPR20129-217 | Abcam | ab219803 | No |
| 34 | IgM | 150Nd | MHM88 | Biolegend | 314527 | No |
| 35 | KLRG-1 | 161Dy | REA261 | MACS | 120-014-229 | No |
| 36 | NKp44 | 147Sm | P44-8 | Biolegend | 325102 | No |
| 37 | NKp44 | 147Sm | 253415 | R&D System | MAB22491 | No |
| 38 | NKp46 | 174Yb | n1D9 | Abcam | ab14823 | No |
| 39 | NKp46 | 174Yb | 9E 2 | Biolegend | 331902 | No |
| 40 | PD-1 | 175Lu | EH 12.2H7 | Fluidigm | 3175008B | No |
| 41 | RORγt | 156Gd | AFKJS-9 | BD eBioscience | 14-6988-82 | No |
| 42 | SLPI | 110Pd | 31 | Abcam | ab17157 | No |
| 43 | Tbet | 149Sm | D6N88 | Fluidigm | 13232BF | No |
| 44 | TCRγδ | 152Sm | B1 | Biolegend | 331202 | No |
| 45 | TCRγδ | 152Sm | 11F2 | Fluidigm | 3152008B | No |
| 46 | TIGIT | 143Nd | MBSA430 | BD eBioscience | 16-9500-82 | No |

**
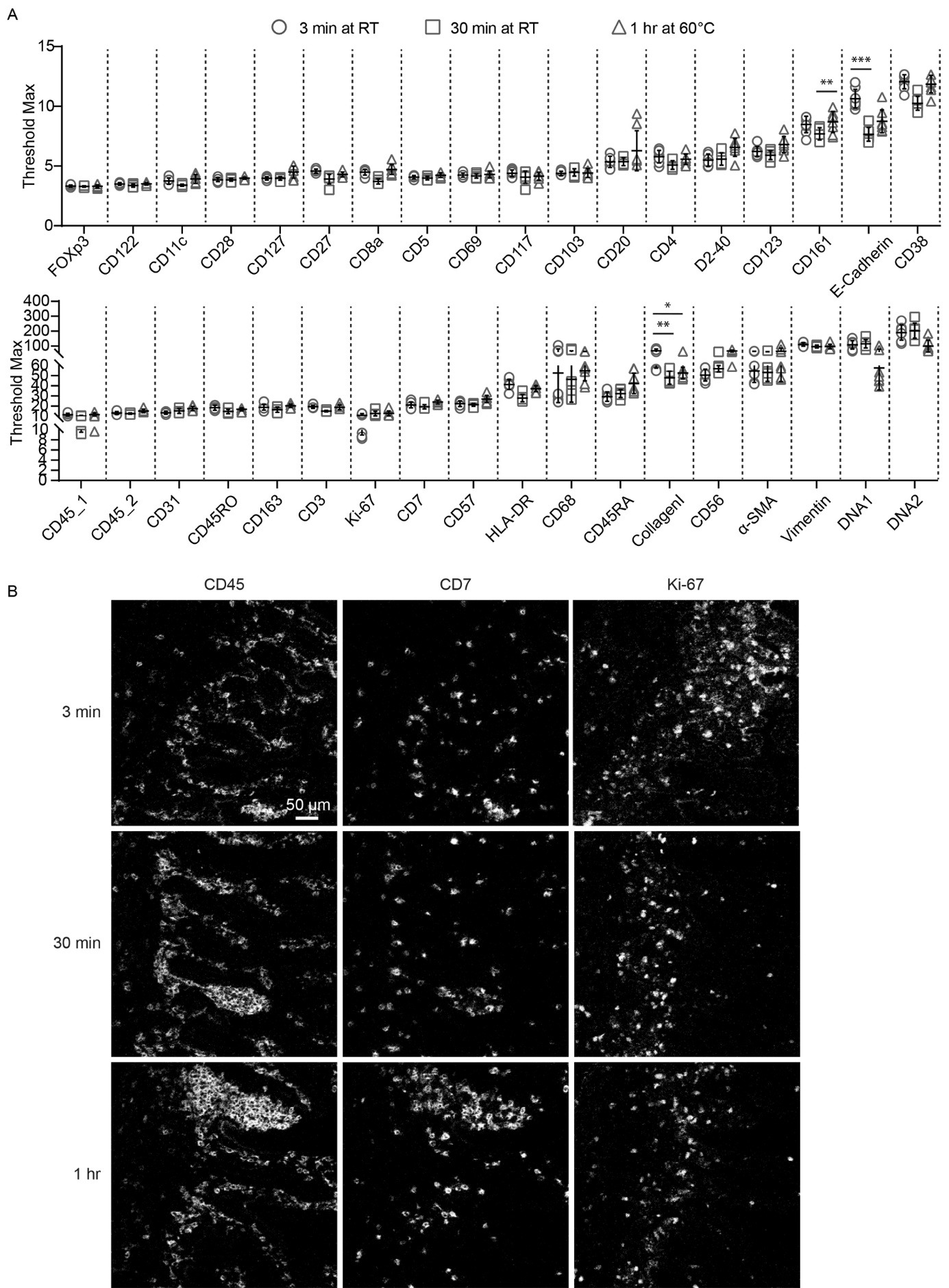
**

**Supplementary figure 1** **Comparison of the impact of the drying time of tissue sections on immune marker performance.** (A) The staining intensity of each antibody for different drying tissue conditions was determined, based on the maximum signal threshold in MCD^TM^ viewer. Black bars indicate median±IQR. Each gray dot represents an individual ROI. *P<0.05 by Kruskal-Wallis test with Dunn’s test for multiple comparisons. (B) The surface markers CD45 and CD3, and the intracellular marker Ki-67 are representative for the variations observed by changing drying time. The minimum signal threshold of 1~2 dual count was set for the immune markers.
